# Methylphenidate Restores Behavioral and Neuroplasticity Impairments in the Prenatal Nicotine Exposure Mouse Model of ADHD: Evidence for Involvement of AMPA Receptor Subunit Composition and Synaptic Spine Morphology in the Hippocampus

**DOI:** 10.3390/ijms23137099

**Published:** 2022-06-26

**Authors:** Darwin Contreras, Ricardo Piña, Claudia Carvallo, Felipe Godoy, Gonzalo Ugarte, Marc Zeise, Carlos Rozas, Bernardo Morales

**Affiliations:** 1 Laboratory of Neuroscience, Faculty of Chemistry and Biology, University of Santiago de Chile, Alameda 3363, Santiago 9170022, Chile; darwin.contrerasp@usach.cl (D.C.); felipe.godoy.g@usach.cl (F.G.); gonzalo.ugarte@usach.cl (G.U.); 2 Departamento de Biología, Facultad de Ciencias Básicas, Universidad Metropolitana de Ciencias de la Educación, Santiago 7760197, Chile; ricardo.pina@umce.cl; 3 Departamento de Ciencias Pedagógicas, Facultad de Educación, Universidad Bernardo O’Higgins, Santiago 8370993, Chile; 4 Centro de investigación e innovación en Gerontología Aplicada (CIGAP), Facultad de Salud, Universidad Santo Tomás, Santiago 8370003, Chile; ccarvallov@santotomas.cl; 5 School of Psychology, Faculty of Humanities, University of Santiago de Chile, Santiago 9170022, Chile; marc.zeise@usach.cl

**Keywords:** ADHD, methylphenidate, hippocampus, LTP, AMPA EPSC

## Abstract

In ADHD treatment, methylphenidate (MPH) is the most frequently used medication. The present work provides evidence that MPH restored behavioral impairments and neuroplasticity due to changes in AMPAR subunit composition and distribution, as well as maturation of dendritic spines, in a prenatal nicotine exposure (PNE) ADHD mouse model. PNE animals and controls were given a single oral dose of MPH (1 mg/kg), and their behavior was tested for attention, hyperactivity, and working memory. Long-term potentiation (LTP) was induced and analyzed at the CA3/CA1 synapse in hippocampal slices taken from the same animals tested behaviorally, measuring fEPSPs and whole-cell patch-clamp EPSCs. By applying crosslinking and Western blots, we estimated the LTP effects on AMPAR subunit composition and distribution. The density and types of dendritic spines were quantified by using the Golgi staining method. MPH completely restored the behavioral impairments of PNE mice. Reduced LTP and AMPA-receptor-mediated EPSCs were also restored. EPSC amplitudes were tightly correlated with numbers of GluA1/GluA1 AMPA receptors at the cell surface. Finally, we found a lower density of dendritic spines in hippocampal pyramidal neurons in PNE mice, with a higher fraction of thin-type immature spines and a lower fraction of mushroom mature spines; the latter effect was also reversed by MPH.

## 1. Introduction

Attention Deficit/Hyperactivity Disorder (ADHD) is the neurodevelopmental disorder with the highest prevalence worldwide [1]; it is characterized by hyperactivity, inattention, and/or impulsivity, affecting learning and sociability at school [2]. The pharmacological treatment of ADHD primarily consists of the administration of psychostimulants, such as amphetamines and, most of all, methylphenidate (MPH), which is used in the ADHD treatment in children and adolescents, as well as in adults nowadays [3]. Furthermore, it is used as a stimulant and nootropic drug by adults and adolescents to improve concentration and intellectual performance. MPH binds to NET (norepinephrine transporters) and DAT (dopamine transporters), inhibiting the reuptake of NE and DA and enhancing their levels in striatum and prefrontal cortex [4]. Indeed, the disorder involves genes that, to a large extent, code for proteins involved in catecholamine transport [5]. However, the mechanisms underlying the MPH-dependent recovery of abnormal behaviors in ADHD and improvement of memory tasks are not completely understood.

### 1.1. The PNE Model of ADHD

Several animal models—including rats, mice, and fish—have been created in order to develop and test new therapies for ADHD in humans [6,7,8,9]. Among non-genetic factors for ADHD, maternal smoking during pregnancy has been reported [10]. Zhu et al. (2012) presented a mouse model for ADHD induced by prenatal nicotine exposure (PNE), displaying hyperactivity, inattention, impulsivity, and working-memory impairment. In this model, oral MPH administration has been shown to be effective alleviating the behavioral abnormalities of this ADHD murine model [11,12].

### 1.2. Role of the Hippocampus in Neuroplastic Changes Related to ADHD and MPH-Induced Neuroplasticity

The PNE model of ADHD involves neuroplastic changes [13,14]. Recently, we have demonstrated in PNE mice displaying ADHD symptoms that atomoxetine, a non-stimulant NET blocker, effectively reestablishes normal behavior and restores impaired neuroplasticity in the hippocampus [15]. If ADHD and MPH action do involve neuroplasticity, it can be expected that, in the hippocampus, arguably the most plastic structure in the CNS [16], plastic changes take place, too. It is at the hippocampal CA3/CA1 synapse where a classical mechanism for LTP induction has been investigated. The postsynaptic site there contains NMDA receptors that, acting as a coincidence detector or an “AND gate”, open when two conditions are fulfilled: liberation of glutamate from the presynaptic site and depolarization at postsynaptic parts. LTP leads to the insertion of new AMPARs into the post-synaptic density, thus increasing synaptic efficacy [17]. 

In the hippocampus, just as in the prefrontal cortex and striatum, MPH also increases NE and DA levels, thus enhancing LTP and LTD [18]. We have demonstrated that, at the hippocampal CA3/CA1 synapse, MPH induces a DA- and NE-receptor-dependent increase of LTP due to the postsynaptic insertion of AMPA receptors (AMPAR) [19]. Furthermore, we have shown that MPH augments hippocampal CA3/CA1 LTP depending on alpha1 and D1/D5 receptor activation via the intracellular messenger chain involving the cAMP/PKA/PSer845 pathway [20]. 

### 1.3. Induction of Hippocampal LTP at the CA3/CA1 Synapse Changes AMPAR Subunit Composition

AMPARs in the postsynaptic membrane exist as homo- or heterotetramers, which are composed of two subunit types. In the basal state, AMPA receptors are present in the postsynaptic density of dendritic spines composed of subunit GluA1/GluA2 and GluA2/GluA3 heteromers and GluA1/GluA1 homomers. LTP induction at the CA3/CA1 synapse is correlated to phosphorylation of the Ser845 at the GluA1 subunit [21]. Induction of LTP results in translocation of subunits changing AMPAR subunit composition and density of AMPARs at the postsynaptic site. GluA2-containing AMPA receptors show linear current vs. voltage relationships in voltage-clamp experiments and are Ca^2+^-impermeable. By contrast, those lacking a GluA2 subunit are Ca^2+^-permeable and exhibit inward rectifying currents [22]. LTP induction implies subunit translocation and fusion to the functional AMPA receptor—correlated to the phosphorylation of residues Ser831, Ser845, and Ser818 at the GluA1 subunits [23]. 

### 1.4. Spine Maturation and ADHD

Because neuroimaging has revealed that, in ADHD patients, the volume in several subcortical structures is reduced, including the hippocampus [24], a brain developmental delay has been proposed as the cause for the disorder [25]. A hallmark in neurodevelopment is the maturation of dendritic spines. Dendritic spines are dynamic structures that are associated with excitatory synapses, exhibiting changes in density, morphology, and functionality during development and activity-dependent remodeling events [26]. Dendritic spines have been classified in five morphological types as long/thin, thin, filopodia, stubby, and mushroom. Filopodia types are immature structures present in neonatal stages and are almost absent in the adult brain, with lifetimes from minutes to hours. Mushroom structures are the largest synaptic contacts and are associated with a functionally mature state (high number of AMPARs), with lifetimes as long as a year [27,28]. Short-term spine dynamics have been reported during awake–sleep cycle [29] and also during high-frequency-stimulation-induced hippocampal LTP [30].

In the present study, we tested neuroplasticity in the PNE mouse model of ADHD and the effects of MPH on neuroplasticity in the hippocampus at behavioral, cellular, and molecular levels. According to our hypothesis, behavioral differences between PNE animals and controls are linked to decreased neuroplasticity, as shown by the induction of LTP. We suggest further that the decreased neuroplasticity is brought forth by postsynaptic changes in AMPAR composition induced by phosphorylation at the Ser845 site of the GluA1 subunit. Furthermore, we intended to demonstrate that corresponding delays in the maturation of dendritic spines accompany changes in synaptic neuroplasticity at electrophysiological and molecular levels. We investigated in the same animal groups the recovery of neuroplasticity induced by a single-dose administration of MPH at all levels.

## 2. Results

### 2.1. A Single Dose of Orally Administered MPH Decreases Motor Hyperactivity and Improves Attention and Working Memory in PNE Mice

To investigate the short-term effects of MPH on behavior in PNE mice, we measured the effect of a single dose—orally administered—of 1 mg/kg of MPH on spontaneous locomotor activity in an open field test (OF) on spatial working memory, using the Y-maze test, and attention, using an object-based attention test (OBA). 

**Figure 1:**
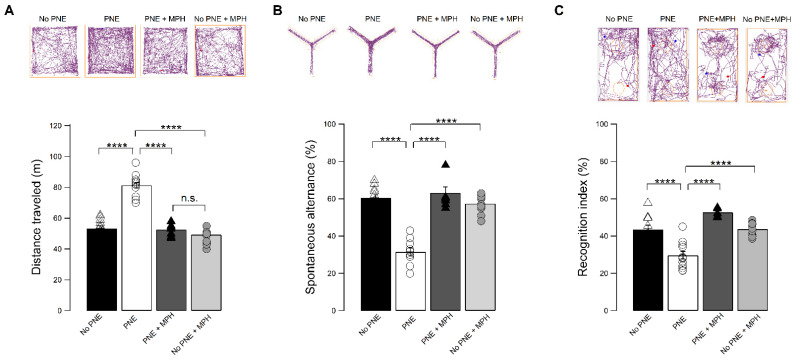
Single MPH administration reverts abnormal behaviors in PNE mice. (**A**) Overall distance traveled (mean ± SEM) for each experimental group in an open field test. PNE mice display enhanced locomotor activity referred to control (no−PNE) mice. (PNE:81.33 ± 1.75 m, n = 15; no−PNE: 53.33 ± 1.0 m, n = 20; two-way ANOVA F_(3,27)_ = 74.41, **** *p* < 0.0001, followed by Tukey post hoc test). MPH-treated PNE and no−PNE mice do not differ in their spontaneous motor activity (no−PNE: 53.332 ± 1.0 m, n = 20; PNE + MPH: 49.26 ± 1.34 m, n = 10, *p* > 0.05). Inset: Sample traces associated with spontaneous locomotor activity for each group of mice during an open field test trial. (**B**) Percentage of spontaneous alternation (mean ± SEM) evaluated in a Y-maze test for the four experimental groups. Spontaneous alternation in PNE mice is about half referred to control no−PNE mice (PNE: 31.50 ± 2.15, n = 10; no−PNE: 60.47 ± 1.70, n = 15; two-way ANOVA F_(3,27)_ = 48.91; **** *p* < 0.0001). MPH-treated PNE and no−PNE mice show no significant difference in their spontaneous alternation (no−PNE: 60.47 ± 1.70, n = 15; PNE + MPH: 57.40 ± 1.58, n = 10; **** *p* < 0.0001). Inset: Sample traces associated to locomotor activity for each group of mice during a Y-maze test trial. (**C**) Plot showing the percentage of recognition index evaluated in object-based attention test for the four experimental groups. The percentage is significantly lower in PNE mice compared to control no−PNE mice (PNE: 29.68 ± 2.07, n = 12; no−PNE: 43.48 ± 2.39, n = 11; two-way ANOVA F_(3,24)_ = 23.65; **** *p* < 0.0001). There is no significant difference in recognition index between MPH-treated PNE mice and no−PNE (PNE + MPH: 43.72 ± 1.17, n = 10; no−PNE: 43.48 ± 2.39, n = 11; *p* > 0.05). Inset: Sample traces associated to locomotor activity for each group of mice during retention phase of OBA test. The red dot inside the chamber area represents the new object.

The upper panels of Figure 1A show locomotor activity traces in the OF test tracked by a zenithal video camera in no−PNE (controls), PNE, MPH-treated PNE, and MPH-treated no−PNE mice. The overall distance traveled during the locomotor activity test was significantly increased in PNE mice compared to no−PNE mice (PNE: 81.33 ± 1.75 n = 15; no−PNE: 53.33 ± 1.01 n = 20; Figure 1A), indicating elevated spontaneous motor activity. After single administration of MPH, the PNE animals exhibited a spontaneous locomotor activity similar to control mice; (PNE + MPH: 49.26 ± 1.34 n = 10; no−PNE: 53.33 ± 1.01 n = 20; Figure 1A). MPH administration in no−PNE mice was without effect.

Figure 1B demonstrates the results of the Y-maze test estimating working memory. Upper panels show sample tracked motor behaviors of no−PNE, PNE, MPH-treated PNE, and MPH-treated no−PNE mice, showing an enhanced activity trough the arms of the device in the PNE mouse. Counts of consecutive alternations (ordinated entries) between arms associated to a working-memory process show that the spontaneous alternation in PNE is significantly decreased compared to no−PNE mice (PNE: 31.50 ± 2.15 n = 10; no−PNE: 60.47 ± 1.70 n = 15, Figure 1B), indicating that MPH restored impaired working-memory function (PNE + MPH: 57.40 ± 1.58 n = 10; no−PNE: 60.47 ± 1.70 n = 15). MPH administration in control animals was without effect.

In Figure 1C, the results of the object-based attention (OBA) assays testing attention are shown. The exploring times for the unknown objects vs. known objects show a low yield for the recognition index. That index was found to be significantly decreased in PNE animals compared to no−PNE mice (PNE: 29.68 ± 2.07 n = 12; no−PNE: 43.48 ± 2.39 n = 11), reflecting inattention for the new object during the test. The impairment in attention was not observed in PNE animals treated with oral MPH, with the recognition index being similar animals of the control group (PNE + MPH: 43.72 ± 1.17 n = 10; no−PNE: 43.48 ± 2.39 n = 11) MPH as no effect on no−PNE recognition index.

Taken together, these results demonstrate that a single oral dose of MPH is effective in counteracting abnormal behaviors associated with ADHD, attenuating hyperactivity, and restoring spatial working and attention. Notably, the single oral administration of MPH did not affect motor activity, working memory, and attention in control (no−PNE) mice. 

### 2.2. MPH Restores Electrophysiological Parameters and Neuroplasticity at the CA3/CA1 Synapse Mainly Acting at Postsynaptic Sites

From the animals tested for their behavioral, hippocampal slices were prepared, electrophysiological recordings were performed, and LTP was induced at the CA3/A1 synapse in order to investigate the correlation between the effects of MPH on behavior and the cellular basis of neuroplastic processes. 

**Figure 2:**
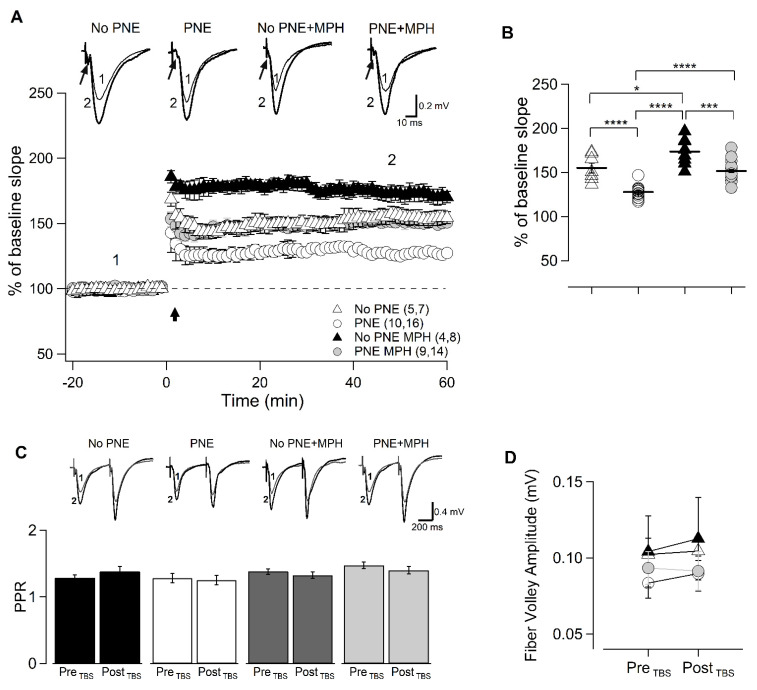
Hippocampal LTP is reduced in PNE animals and restored after single MPH administration. (**A**) Normalized fEPSP slope measurements before and after LTP induction (arrow) in hippocampal slices from no−PNE, MPH-treated no−PNE, PNE, and MPH−treated PNE mice. Inset: Superimposed traces of fEPSP recorded 10 min before (1) and 50 min after (2) TBS for the corresponding experimental groups. Arrows indicate fiber volley preceding postsynaptic signals. (**B**) Scatter plot of normalized fEPSP slope measurement evaluated 50 min after application of TBS (marked 2) during LTP protocol. Hippocampal LTP is significantly lower in PNE mice compared to control no−PNE mice (PNE: 127.8 ± 1.75, n = 10.15; no−PNE: 155.1 ± 5.58, n = 10.12, one−way ANOVA followed by Tukey post hoc test, F_(3,41)_ = 30.06; **** *p* < 0.0001). However, LTP in MPH−treated PNE animals is significantly higher than in untreated PNE mice (PNE: 127.8 ± 1.75, n = 10.15; PNE + MPH: 151.6 ± 3.246, n = 9.14, * *p* < 0.05; *** *p* < 0.01; **** *p* < 0.001) (**C**). P2/P1 ratio (PPR) measurements obtained by paired pulse protocol before and after induction of LTP by TBS in brain slices taken from the four experimental groups. There are no significant differences in the ratio before and after LTP induction from no−PNE, MPH−treated no−PNE, PNE, and MPH−treated PNE mice, nor are there significant differences amongst the groups (PNE_(preTBS)_: 1.29 ± 0.069, PNE_(post TBS)_: 1.26 ± 0.073; no−PNE_(preTBS)_: 1.287 ± 0.04; no−PNE_(postTBS)_: 1.381 ± 0.075; no−PNE + MPH_(preTBS)_: 1.394 ± 0.04; no−PNE + MPH_(postTBS)_: 1.338 ± 0.05; PNE + MPH_(preTBS)_: 1.486 ± 0.05; PNE + MPH_(postTBS)_: 1.413 ± 0.06; *p* > 0.05). Inset: Superimposed traces of fEPSPs recorded during the paired-pulse protocol before (1) and after TBS (2) in brain slices from the four experimental groups. (**D**) Presynaptic fiber volley amplitude before and after induction of LTP by TBS in slices from no−PNE (open triangles), PNE (open circles), MPH−treated no−PNE (black triangles), and MPH-treated PNE mice (gray circles). There is no significant difference in the volley amplitude before and after LTP induction in the four groups, nor are there significant differences among the four experimental groups (PNE_(preTBS)_: 0.084 ± 0.01 mV, PNE_(post TBS)_: 1.26 ± 0.073; no−PNE_(preTBS)_: 0.010 ± 0.01 mV, no−PNE_(post TBS)_: 1.105 ± 0.01 mV; PNE + MPH_(preTBS)_: 0.093 ± 0.02 mV, PNE + MPH_(post TBS)_: 0.091 ± 0.013 mV; no−PNE + MPH_(preTBS)_: 0.104 ± 0.02 mV, PNE_(post TBS)_: 0.113 ± 0.023 mV). The values given in parentheses correspond to number of animals and recorded slices, respectively.

As described in the Materials and Methods, fESPs were recorded and LTP was induced. Upper traces of Figure 2A show superimposed fEPSP recordings before and after LTP induction obtained in slices from the four groups of mice: no−PNE, PNE, MPH-treated no−PNE, and MPH-treated PNE. Measuring the slope of the onset of fEPSPs revealed that TBS-induced hippocampal LTP in brain slices prepared from PNE mice is severely reduced. As shown in the plot included in Figure 2B, the amplitude of hippocampal LTP in PNE mice is reduced to about 50% of the value recorded in slices taken from no−PNE animals (PNE: 127.8 ± 1.75 n = 10.15; no−PNE: 155.1 ± 5.58 n = 10.12). However, PNE animals treated with a single oral dose of MPH show a complete recovery of LTP (PNE: 127.8 ± 1.75 n = 10.15; PNE + MPH: 151.6 ± 3.246 n = 9.14). As we have previously reported [20], a single dose of MPH increases hippocampal LTP in control no−PNE by about 50%. 

To determine whether the recovery of LTP by MPH was due to presynaptic or rather postsynaptic changes, we used a paired-pulse facilitation protocol that allows us to study the facilitation of synaptic response as the ratio of onset slopes of first and second evoked responses. Bar plots of Figure 2B show that this ratio has similar values in PNE and no−PNE slices (PNE_(preTBS)_: 1.29 ± 0.069, PNE_(post TBS)_: 1.26 ± 0.073; no−PNE_(preTBS)_: 1.287 ± 0.04; no−PNE_(postTBS)_: 1.381 ± 0.075). In addition, the ratio is equivalent in no−PNE and PNE animals treated with oral MPH (no−PNE + MPH_(preTBS)_: 1.394 ± 0.04; no−PNE + MPH_(postTBS)_: 1.338 ± 0.05; PNE + MPH_(preTBS)_: 1.486 ± 0.05; PNE + MPH_(postTBS)_: 1.413 ± 0.06). In addition, Figure 2D shows that the amplitude of the presynaptic volley (action potentials at the presynaptic-terminals-associated signal) preceding the CA3/CA1-dependent field synaptic signals in hippocampal slices taken from MPH-treated PNE and untreated PNE mice remains almost unchanged (PNE_(preTBS)_: 0.084 ± 0.01 mV, PNE_(post TBS)_: 1.26 ± 0.073; PNE + MPH_(preTBS)_: 0.093 ± 0.02 mV, PNE + MPH_(post TBS)_: 0.091 ± 0.013 mV). 

Both the paired-pulse facilitation and comparison of presynaptic fiber volleys provide evidence that presynaptic components of the CA3/CA1 synapse are not involved in the MPH-dependent recovery of hippocampal LTP in PNE mice.

### 2.3. AMPAR-Dependent EPSCs Are Decreased in CA1 Pyramidal Neurons from PNE Mice and Are Restored in PNE Animals after Administration of MPH

**Figure 3:**
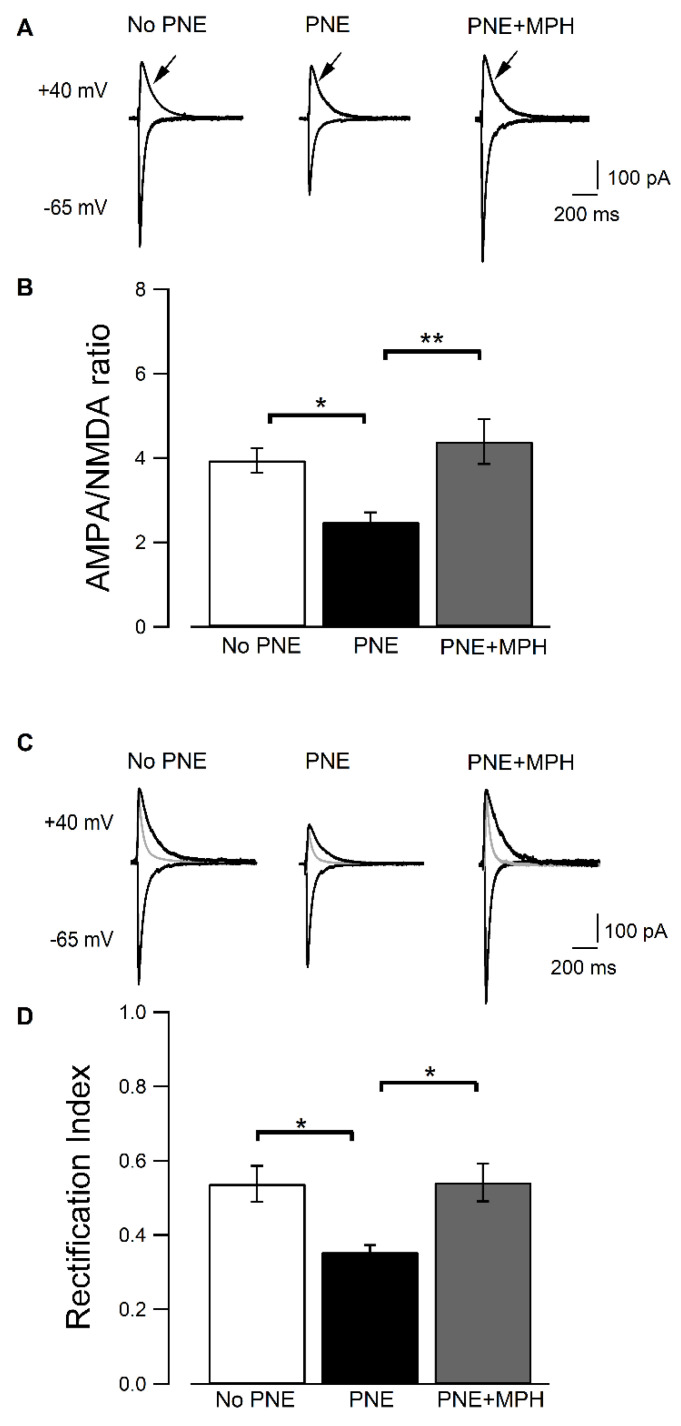
AMPAR EPSCs are reduced in hippocampal neurons of PNE mice with a decrease in the rectification index and restored to normal by MPH. Whole−cell voltage clamp recordings. (**A**) EPSCs measured at +40 and −65 mV in pyramidal neurons contained in brain slices from no−PNE, PNE, and MPH-treated PNE mice, arrow indicated NMDA component. (**B**) Ratios of EPSC amplitudes generated by AMPARs vs. those of NMDARs. That ratio is significantly reduced in PNE neurons (no−PNE: 3.945 ± 0.29, n = 5.13; PNE: 2.483 ± 0.23, n = 7.15, * *p* < 0.05) compared to no−PNE neurons and restored by MPH (no−PNE: 3.945 ± 0.29, n = 5.13; PNE + MPH: 4.387 ± 0.53, n = 4, ** *p* < 0.01; one-way ANOVA F_(2,24)_ = 6.53 followed by Turkey post hoc test). (**C**) AMPAR EPSCs (recorded after perfusion with 5 µM AP-5 to suppress NMDAR-generated currents) measured at +40 and −65 mV in pyramidal neurons from no−PNE, PNE, and MPH-treated PNE mice. Gray traces: Outward EPSCs before perfusion with AP-5. (**D**) Rectification indices of AMPAR EPSCs recorded in pyramidal neurons from no−PNE, PNE, and MPH-treated PNE mice. The rectification index is significantly lower in PNE neurons (PNE: 0.354 ± 0.019, n = 5.11; no−PNE: 0.538 ± 0.048, n = 6.9; * *p* < 0.05) compared to no−PNE neurons, whereas in neurons from MHP-treated PNE mice, it is significantly different from PNE mice, but almost identical to the one in controls (PNE: 0.354 ± 0.019, n = 5.11; PNE + MPH: 0.542 ± 0.051, n = 4.7; no−PNE: 0.538 ± 0.048, n = 6.9; * *p* < 0.05; one-way ANOVA F_(2,21)_= 5.39 followed by Turkey post hoc test).

Next, we measured the AMPAR− and NMDAR−dependent EPSCs in CA1 pyramidal neurons, using whole-cell patch-clamp recordings. In Figure 3A, EPSCs recorded at −65 mV and +40 mV from slices of controls (no−PNE), PNE, and PNE + MPH mice are shown. The AMPAR-dependent current measured at −65 mV is significantly decreased in pyramidal neurons from PNE mice compared to no−PNE neurons and restored to normal following MPH-treatment. AMPA currents are significantly reduced in hippocampal neurons from PNE mice compared to the control (no−PNE) group (no−PNE: 841.7 ± 59.44 pA, n = 5.8; PNE: 635.3 ± 22.05 pA, n = 6.9; PNE + MPH: 959.2 ± 51.26 pA, n = 4.6; not shown). In Figure 3B AMPAR/NMDAR current ratios are displayed. The ratio is decreased for neurons derived from PNE animals compared to those from no−PNE animals, essentially because of AMPAR current reduction. In neurons from MPH-treated PNE mice the AMPAR/NMDAR currents ratio is similar to controls (no−PNE: 3.945 ± 0.29, n = 5.13; PNE: 2.483 ± 0.23, n = 7.15; PNE + MPH: 4.387 ± 0.53, n = 4.7).

### 2.4. Induction of LTP, Its Reduction in PNE Mice and Recovery by MPH Is Correlated to Changes in AMPAR Subunit Composition

During development, molecular changes occur affecting hippocampal AMPA receptors, such as editing and changes in subunit composition of surface receptors [31]. It has been reported that, in active synapses, GluA1:GluA2 heteromers (Ca^2+^-impermeable and linear I–V relationship) and GluA1:GluA1 homomers (Ca^2+^-permeable and inward rectifying) can be found [32]. To investigate possible changes in the subunit composition of AMPA receptors in PNE-mice-derived pyramidal neurons, we analyzed the EPSCs in the presence of the selective blocker of NMDARs, AP-5, to isolate AMPAR-generated currents. As the rectification index of EPSCs is characteristic for AMPAR composition, the rectification indices of AMPAR EPSCs were calculated. Outward AMPAR currents (recorded at +40 mV) of neurons from PNE mice were found to be considerably reduced compared to currents recorded in no−PNE-derived neurons (Figure 3D). Thus, the rectification index of AMPAR currents (ratio of currents recorded at +40 mV vs. −65 mV) is decreased in PNE-derived pyramidal neurons compared to no−PNE neurons (PNE: 0.354 ± 0.019, n = 5.11; no−PNE: 0.538 ± 0.048, n = 6.9; * *p* < 0.05; Figure 3D). In PNE animals treated with MPH, the value for the rectification index is recovered to values near those estimated for AMPAR currents recorded in no−PNE control animals (PNE: 0.354 ± 0.019, n = 5.11; PNE + MPH: 0.542 ± 0.051, n = 4.7; * *p* < 0.05). 

### 2.5. MPH Administration Restores Phosphorylation of Ser845 of the GLUA1 Subunit and Surface GluA1 AMPA Receptor Levels in CA1 Pyramidal Neurons of PNE Mice

Several phosphorylation events occur in AMPA receptors induced by TBS during LTP development. It has been documented that, during hippocampal LTP, CaMKII/PKC-dependent and PKA-dependent phosphorylation of residues Ser845 and Ser831 (among others) occur at the GluA1 subunit of AMPA receptors, associated to the translocation and fusion of receptor-containing vesicles into the postsynaptic membrane [20]. 

**Figure 4:**
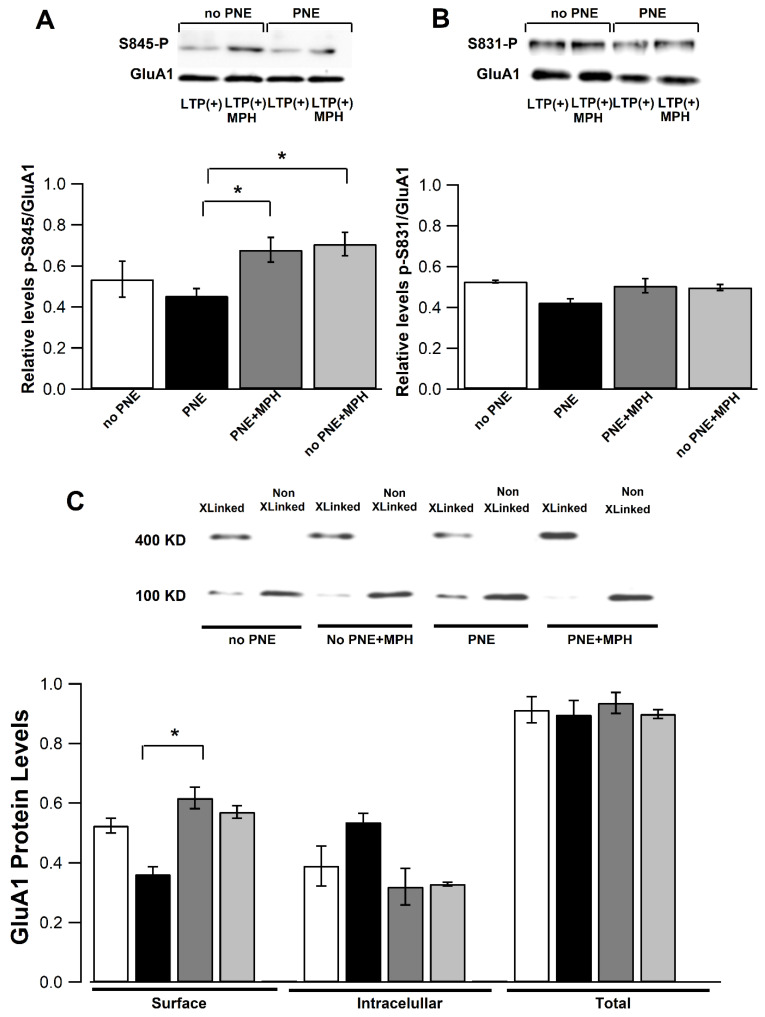
LTP-associated phosphorylation of Ser845 residues at the GluA1 subunit of AMPA receptors and surface fraction of GluA1-containing receptors are decreased in PNE animals and are recovered after MPH administration. (**A**) Western blot for phospho-Ser845 of GluA1 subunit of AMPA receptor in protein extracts of CA1 areas (after LTP induction) from brain slices of no−PNE (lane 1), MPH-treated no−PNE (lane 2), PNE (lane 3), and MPH-treated PNE animals (lane 4). Bar plot: Densitometric quantification of phospho-Ser845-GluA1 reactive bands normalized to the band total GluA1 associated band for each condition. Phosphorylation of Ser845 residues of GluA1 subunits is significantly lower in samples of CA1 areas from PNE mice compared to those obtained in samples from control (no−PNE) mice; n PNE mice treated with oral MPH phosphorylation levels were found to be restored (no−PNE: 0.535 ± 0.009, n = 3.3; PNE: 0.454 ± 0.035, n = 3.3; PNE + MPH: 0.679 ± 0.060 n = 3.3, * *p* < 0.05). (**B**) Same as in (**A**), but for phospho-Ser831 of AMPA receptor GluA1 subunits (after LTP induction) from no−PNE (lane 1), MPH-treated no−PNE (lane 2), PNE (lane 3), and MPH-treated PNE animals (lane 4). Bar plot: Densitometric quantification of phospho-Ser831-GluA1 reactive bands. Phosphorylation of Ser831 residue of GluA1 subunit is not statistically different among all four samples (no−PNE: 0.527 ± 0.007, n = 3.3; no−PNE + MPH: 0.499 ± 0.015, n = 3.3; PNE: 0.424 ± 0.019, n = 3.3; PNE + MPH: 0.507 ± 0.035, n = 3.3, *p* > 0.05) (**C**) Western blot for AMPAR GluA1 subunits in protein extracts of paired BS^3^-crosslinked and non-crosslinked CA1 areas (after LTP induction) from brain slices of no−PNE (lanes 1 and 2), MPH-treated no−PNE (lanes 3 and 4), PNE (lanes 5 and 6), and MPH-treated PNE animals (lanes 7 and 8). Bar plot: Densitometric quantification of reactive bands for surface (~400 kDa reactive band) and intracellular pools (~100 kDa reactive band) of the GluA1 subunit in crosslinked samples, and total amount of GluA1 subunits evaluated in paired non-crosslinked samples for each experimental condition. Surface fraction of GluA1 subunits is significantly lower in PNE mice compared to samples from no−PNE mice (no−PNE: 0.524 ± 0.025, n = 3.3; PNE: 0.361 ± 0.025, * *p* < 0.05). The decrease seen in PNE mice is completely restored after administration of MPH (PNE: 0.361 ± 0.025 vs. PNE-MPH: 0.617 ± 0.036, * *p* < 0.05). Consistently, in CA1 samples from MPH-treated PNE mice, the intracellular pool of GluA1 subunit appears to be lower compared to samples from PNE mice without, however, reaching significance. The total amount of GluA1 subunits is not statistically different among all four samples (non-crosslinked) (PNE: 0.536 ± 0.029, n = 3.3; PNE-MPH: 0.32 ± 0.061, * *p* < 0.05).

We performed Western blots to determine the phosphorylation states of Ser845 and Ser831 residues in the GluA1 subunit in the CA1 area of PNE mice and control mice. The LTP-induced phosphorylation of the Ser845 residue of the GluA1 subunit was not found to be decreased significantly in CA1 areas taken from PNE animals compared to the phosphorylation level in samples obtained from CA1 areas of no−PNE mice (Figure 4A). However, MPH administration in PNE mice caused a significant increase in LTP-dependent phosphorylation of the Ser845 site (expressed as ratio of phosphoylated-Ser845/total GluA1; Figure 4A; no−PNE: 0.535 ± 0.009, n = 3.3; no−PNE + MPH: 0.706 ± 0.057, n = 3.3; PNE: 0.454 ± 0.035, n = 3.3; PNE + MPH: 0.679 ± 0.060 n = 3.3). In contrast, the LTP-dependent phosphorylation of Ser831 was not modified after treatment with MPH treatment in PNE and no−PNE mice (no−PNE: 0.527 ± 0.007, n = 3.3; no−PNE + MPH: 0.499 ± 0.015, n = 3.3; PNE: 0.424 ± 0.019, n = 3.3; PNE + MPH: 0.507 ± 0.035, n = 3.3; Figure 4B).

To estimate the density of AMPA receptors in the surface of pyramidal neurons in PNE mice and the effect of MPH on this distribution, we performed a crosslink assay, using the membrane impermeable BS^3^ crosslinker agent. The exposure of CA1 slices to BS3 at 4 °C induces the generation of high-molecular complexes by covalent binding between near AMPA subunits present in the postsynaptic membrane (surface fraction). The Western blots in Figure 4C show that protein extracts of CA1 areas obtained from PNE mice contain significantly lower amounts of GluA1-containing AMPA receptors at the cell surface (high-molecular-weight band corresponding to crosslinked receptors in lanes 1 and 5) after the LTP protocol compared to no−PNE samples (no−PNE: 0.524 ± 0.025, n = 3.3; PNE: 0.361 ± 0.025, * *p* < 0.005). This analysis also shows that the protein sample of CA1 areas from PNE mice treated with oral MPH have significantly higher levels of AMPA receptors at the cell surface compared to samples of untreated PNE mice (lanes 5 and 7 in Western blot; PNE: 0.361 ± 0.025 vs. PNE-MPH: 0.617 ± 0.036). Even though the difference between PNE and control did not reach significance, the MPH-induced increase in the surface fraction of GluA1-containing AMPA receptors is higher in PNE mice compared to treated no−PNE mice (lines 3 and 7). This enhancement of surface receptors induced by MPH is associated with lower levels of intracellular pool of receptors (monomeric GluA1 subunit-associated band) in treated PNE mice (PNE: 0.536 ± 0.029, n = 3.3; PNE-MPH: 0.32 ± 0.061, lanes 3 and 7), suggesting that the mobilization process of receptors to the surface is enhanced in MPH-treated PNE mice compared to treated no−PNE animals. Altogether, the results suggest that PNE mice have a lower amount of GluA1-containing AMPA receptors in the plasma membrane of pyramidal neurons associated to lower phosphorylation of the Ser845 of GluA1 subunit. This molecular evidence is consistent with lower amplitudes of AMPA-dependent EPSC recorded in pyramidal cells by using patch-clamp, as shown in Figure 3. Importantly, both the phosphorylation of Ser845 and insertion of functional AMPA receptors in the plasma membrane of pyramidal cells can be enhanced (restored) by a single dose of MPH. 

### 2.6. Dendritic Spine Density and Maturation State Are Impaired in PNE Mice; MPH Restores Maturation of Dendritic Spines, but Not Their Density

**Figure 5:**
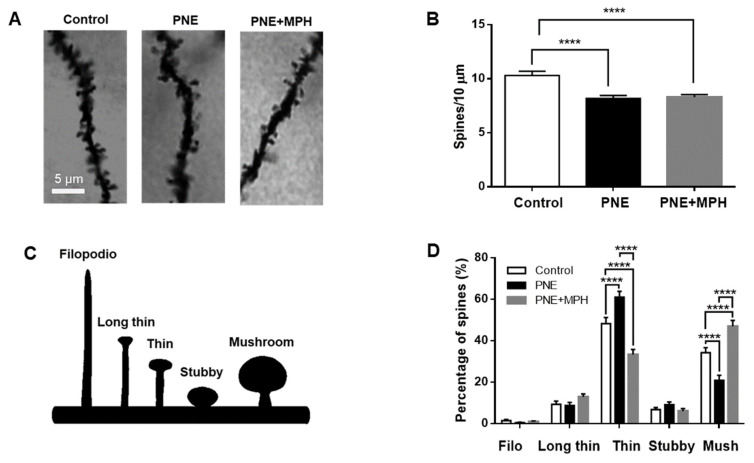
Density of dendritic spines in hippocampal neurons is reduced in PNE animals with a significant increase in fraction of immature thin-type spines and reduction of mature mushroom-type spines. MPH administration restores maturation of spines, but not density. (**A**) Microphotographs of spines on dendrites of Golgi-stained pyramidal neurons of the CA1 area of hippocampal sections from no−PNE (control), PNE, and MPH-treated PNE mice (PNE + MPH). (**B**) Bar plot: Dendritic spine density (per 10 μm) in CA1 pyramidal neurons of no−PNE, PNE, and MPH-treated hippocampal slices. PNE animals exhibit significantly lower spine density compared to control (no−PNE) mice (PNE: 8.18 ± 0.272, n = 824 vs. control: 10.03 ± 0.389, n = 581 **** *p* ≤ 0.0001). Spine densities in PNE and MPH-treated PNE mice are not significantly different (PNE: 8.18 ± 0.272, n = 824 vs. MPH-treated PNE: 8.29 ± 0.232, n = 622, *p* > 0.05, one-way ANOVA with post hoc Tukey Test). (**C**) Schematic representation of morphological classification of dendritic spines. Left-right sequence represents maturation states of spines during brain development. (**D**) Bar plot: Quantification of different types of dendritic spines (filopodia, long/thin, thin, stubby, and mushroom) in pyramidal neurons contained in hippocampal slices from control, PNE, and MPH-treated PNE mice. The percentage of thin-type spines is significantly higher in PNE neurons compared to control neurons (PNE: 61.0 ± 2.822% vs. control: 48.23 ± 2.947%, **** *p* ≤ 0.0001). By contrast, the fraction of mushroom-type spines is significantly lower in PNE neurons compared to control neurons (PNE: 20.8 ± 2.517% vs. control: 34.25 ± 2.411%, **** *p* < 0.0001). CA1 hippocampal neurons of MPH-treated PNE mice exhibit a lower fraction of thin-type spines compared to neurons contained in PNE slices (MPH-treated PNE: 33.27 ± 2.479% vs. PNE: 61.0 ± 2.822%, **** *p* < 0.0001) and a higher fraction of mushroom-type spines (MPH-treated PNE: 46.90 ± 2.875% vs. PNE: 20.8 ± 2.517%, **** *p* < 0.0001). The percentages of filopodia, long/thin, and stubby-type spines are not significantly different in neurons contained in hippocampal slices derived from control, PNE, and MPH-treated PNE mice (two-way ANOVA with post hoc Tukey test).

It is well-known that, during brain development, neuroplastic processes at the synaptic level occur, changing the density and morphology of dendritic spines correlated to the maturation of synaptic transmission efficacy [33]. To determine the density and morphology of dendritic spines in hippocampal pyramidal neurons, we performed Golgi staining with the corresponding quantifications. Figure 5A shows microphotographs of representative dendritic segments at hippocampal pyramidal neurons from control and PNE mice. Quantification of dendritic spine density reveals a significant decrease of spine density in PNE animals (PNE: 8.18 ± 0.272, n = 824 vs. no−PNE: 10.03 ± 0.389, n = 581 **** *p* < 0.0001; Figure 5B. Spine density in PNE and MPH-treated PNE mice are not significantly different (PNE: 8.18 ± 0.272, n = 824 vs. MPH-treated PNE: 8.29 ± 0.232, n = 622; one-way ANOVA with post hoc Tukey Test).

The morphology of dendritic spines was correlated to functional states of maturation during neural development. Five types of spines were identified, considering the ratio between head and tail substructures (Figure 5C). Figure 5D shows the analysis and quantification of spine morphology in hippocampal CA1 neurons from control and PNE mice. In PNE mice, thin-type spines are increased in hippocampal neurons in slices obtained from PNE mice compared to those from non-PNE (PNE: 61.0 ± 2.822% vs. no−PNE: 48.23 ± 2.947%, **** *p* < 0.0001). By contrast, the mushroom-type spines (classified as mature spines) are decreased in the neurons of PNE mice compared to no−PNE mice (PNE: 20.8 ± 2.517% vs. no−PNE: 34.25 ± 2.411%, **** *p* < 0.0001). Considering that the thin-type spines are associated with immature structures and the mushroom-type spines are associated to mature spines, these results suggest that dendritic spine development in pyramidal neurons is delayed in PNE mice. In addition, we analyzed the effect of a single dose of MPH (1 mg/kg, oral) over the dendritic spines of CA1 hippocampal neurons of PNE mice. Figure 5D shows that, after three hours of oral administration, the CA1 neurons of MPH-treated PNE mice contain a significantly lower fraction of immature thin-type spines compared to untreated PNE animals (MPH-treated PNE: 33.27 ± 2.479% vs. PNE: 61.0 ± 2.822%, **** *p* < 0.0001), even below the values observed in control mice. On the other hand, the fraction of mushroom-type spines is significantly enhanced in MPH-treated PNE mice compared to untreated PNE animals (MPH-treated PNE: 46.90 ± 2.875% vs. PNE: 20.8 ± 2.517%, **** *p* < 0.0001) and larger than the fraction of mushroom-type spines observed in control mice. These results suggest that MPH restores the maturation state of dendritic spines in PNE animals without changing synapse densities during a time window of three hours.

## 3. Discussion

### 3.1. Restoration of ADHD Symptoms to Normal by Single Oral Administration of MPH

This work yields novel data about MPH action on the PNE model for ADHD. PNE animals display the behavioral impairments of hyperactivity, lower working memory, and inattention, as is in line with the behavioral characterization of the PNE mouse model reported by Zhu et al. [11,34] and similar to the symptoms seen in human ADHD patients. A single oral administration of MPH in PNE animals restored motor activity, working memory, and attention evaluated in the OF, Y-maze, and OBA tests, respectively. Considering that the effect of MPH on behavior of the PNE mice was observed 90 min after administration (see Methods) a short-term modulation of synaptic mechanisms involved in cognitive processes such as working memory can be proposed. In contrast, no significant MPH effect on the behavior of control (no−PNE) animals was observed, and this is in concordance with Zhu et al. [12]. The dose used (1 mg/kg) is well within the therapeutic range for the treatment of children. It is not easy to demonstrate, and the literature is somewhat ambiguous about an improvement of learning tasks and other cognitive functions at 1 mg/kg in rodents [35,36]. It may well be that the effect is relatively small and may not have been detected in our behavioral tests. By all means, the MPH-induced improvement compared to PNE animals was highly significant.

In hippocampal slices, PNE mice exhibit a significant reduction in hippocampal TBS-induced LTP, in line with data we have reported recently [15]. MPH administration in PNE animals restores hippocampal LTP to levels similar to the ones recorded in slices from control (no−PNE) mice. MPH also enhances TBS-induced LTP in slices from control (no−PNE) mice, as described previously [20]. Experiments employing perfusion of MPH on brain slices had no effect on baseline fEPSPs recorded before TBS-dependent induction of LTP (data not shown), suggesting that the effect of MPH on LTP is related to the neuroplastic processes involved in the induction of hippocampal LTP. 

### 3.2. Differences in Neuroplasticity between PNE Mice and Controls and Restoration of Neuroplasticity by MPH Are Essentially Due to Postsynaptic Processes

In line with our previous findings [19,20], the paired-pulse facilitation (P2/P1 ratio) was not modified during LTP induction in slices from MPH-treated PNE mice. Furthermore, the presynaptic fiber volley amplitude was not modified either after TBS-induced LTP in slices from MPH-treated PNE animals (Figure 2). This finding suggests that presynaptic components are not involved in the neuroplastic effect of MPH at the CA3/CA1 synapse and, therefore, that it does not involve recruitment of additional terminals during LTP induction.

### 3.3. Electrophysiological and Molecular Evidence for Differences/Changes in AMPAR Composition

The whole-cell recordings demonstrate that amplitudes of the AMPAR EPSCs (at −65 mV) in CA1 pyramidal neurons in slices from PNE mice are smaller than the ones recorded in control neurons, whereas amplitudes of NMDAR EPSCs (at +40 mV) are not affected (Figure 3A,B). Our analysis of AMPAR EPSCs reveals a lower rectification index in hippocampal neurons from PNE mice as compared to controls (Figure 3D). In this context, it has been shown that GluA2-containing AMPARs are calcium impermeable, with a rectification index close to 1.0. By contrast, GluA2-lacking receptors are calcium permeable and exhibit inward rectification [31]. Thus, the lower rectification index in PNE mice indicates more inward rectification, and, thus, more insertion of GluA2-containing receptors in hippocampal neurons of PNE mice and a lesser proportion of GluA1/GluA1 AMPARs, lacking GluA2. MPH treatment restored amplitudes and rectification indices to control levels (Figure 3). Several phosphorylation events occur in AMPA receptors induced by TBS during LTP development. It has been documented that, during hippocampal LTP, the CaMKII/PKC-dependent and PKA-dependent phosphorylation of residues Ser845 and Ser831 (among others) occurs at the GluA1 subunit of AMPA receptors, associated to translocation and fusion of receptor-containing vesicles into the postsynaptic membrane [20]. The electrophysiological evidence mentioned above is consistent with our measurements of the phosphorylation state of the Ser845 residue of GluA1 subunit involved in the LTP-induced insertion of AMPARs (Figure 4). Western blots from CA1-region samples taken from PNE animals indicate that the LTP-dependent phosphorylation of Ser845 is decreased compared to samples from control animals, with no significant changes in the phosphorylation of Ser831 residue, as is consistent with the results of the crosslink assay estimating the pool of GluA1-containing AMPA receptors at the cell surface. The latter experiments show that CA1 samples from PNE animals contain lower levels of these receptors at the cell surface, with a significant increase of the intracellular pool of non-mobilized GluA1-containing AMPARs (Figure 4C). The assay also indicates that the total amount of GluA1 protein levels is not changed in CA1 samples from PNE mice compared to those from no−PNE CA1 samples, suggesting that only the subcellular fraction of preformed AMPA receptors is altered in PNE mice.

Our findings are consistent with former ones: LTP induction implies subunit translocation and fusion to functional AMPA receptor—correlated to the phosphorylation of residues Ser831, Ser845, and Ser818 at the GluA1 subunits [23]. The phosphorylation of Ser845 residues by PKA has been associated with the trafficking of vesicles to extrasynaptic membrane parts during LTP and is increased by the activation of adrenergic receptors [37,38]. Thus, AMPA-containing vesicles are translocated and inserted into extrasynaptic membrane domains, and then, by lateral diffusion, they become anchored in the postsynaptic density by the scaffold protein PDS-95 and transmembrane AMPARs’ regulatory proteins (TARPs) [39].

In the light of the results described above, we suggest that MPH may promote the insertion of GluA1/GluA1-containing AMPARs at the cell surface, increasing the AMPAR EPSCs, recovering TBS-induced LTP, and, consequently, causing an improvement in working memory and attention in PNE mice.

### 3.4. Maturation of Dendritic Spines Is Correlated to the Differences/Changes Observed with Electrophysiological and Molecular Methods

Previous reports have demonstrated that the density of AMPA and NMDA receptors is proportional to spine volume [40,41]. In particular, mushroom spines with large PSDs contain more AMPA receptors, making these synapses stronger [42,43]. Thus, hippocampal LTP increases spine-head size [44,45,46,47], accompanied by an accumulation of AMPA receptors at the PSD [46]. Furthermore, Vyazovskiy [48] have shown that, during wakefulness, the phosphorylation of Ser831 in GluA1-containing AMPA receptors is observed, as in hippocampal LTP induction, whereas, during sleep, activation of CaMKII and dephosphorylation of Ser845 are observed, similar to changes that occur during LTD induction. In addition, using uncaged glutamate and calcium imaging in whole-cell recordings revealed that nascent spines are coupled to the maturation of glutamatergic synapses [49]. 

Our morphological study of the effects of MPH on dendritic spines at pyramidal neurons in brain slices derived from PNE mice suggests a fast remodeling of dendritic spines, albeit not a creation of new spines, since spine density remained unchanged by MPH. As only three hours elapsed between the administration of MPH and animal sacrifice, there was a very rapid boost of mushroom-type spines, and this is associated with the mature phenotype (Figure 5). The above findings demonstrate for the first time that, in the PNE model of ADHD, spine maturation is delayed. MPH fully reverts that delay in three hours or less, probably through a mechanism that involves insertion of AMPARs in the PSDs augmenting the share of GluA1-containing AMPARs. Our results also confirm the developmental character of ADHD. It will be interesting to investigate how the molecular changes are related to the morphological ones. We hypothesize that insertion of new AMPARs is coupled to an expansion of spine areas. 

## 4. Conclusions

In the present study, using the prenatal nicotine exposure (PNE) murine model for ADHD, we demonstrated that a single oral dose of MPH can restore the abnormal ADHD-associated behaviors of hyperactivity, impaired working memory, and inattention (Figure 1). We further presented electrophysiological evidence that MPH reestablishes normal hippocampal LTP in PNE mice (Figure 2), enhancing AMPAR-dependent EPSCs in hippocampal pyramidal neurons (Figure 3). We also demonstrated that this effect is correlated to the insertion of preformed AMPA receptors into the surface of the postsynaptic membrane, associated to the phosphorylation of Ser845 of the GluA1 subunit of AMPA receptors (Figure 4). Finally, we obtained evidence that, in pyramidal CA1 neurons of PNE mice, the maturation of dendritic spines is delayed; that is, the number of mushroom-type mature spines is reduced. Single oral MPH administration in PNE mice rapidly restores the maturation status (Figure 5). Taken together, our results demonstrate that MPH administered to PNE mice restores ADHD symptoms to normal, bringing back the magnitude of hippocampal LTP by insertion of preformed AMPA receptors retained in the intracellular pool into the postsynaptic membrane. Furthermore, the changes observed are correlated with the delayed or restored maturation of dendritic spines, respectively.

## 5. Materials and Methods

### 5.1. Management Protocol and Animal Care

C57BL/6 mice were maintained in light/dark 12 h cycles, with food and water ad libitum, at humidity/temperature-controlled conditions. The experimental protocols and the animal-handling procedures used in this work were approved by the ANID (National Research and Development Agency) and the Bioethics Committee of the University of Santiago of Chile. 

### 5.2. Murine Model of ADHD Induced by Prenatal Nicotine Exposure 

The ADHD animals were obtained following the protocol described by Zhu [11,34]. Female C57BL/6 mice were treated with 0.1 mg/mL nicotine (concentration to which is obtained a maximal number of offspring with ADHD symptoms) during 3 weeks before mating and during the whole period of pregnancy. Under these conditions, nearly 80% of the offspring of mothers treated with prenatal nicotine showed an ADHD phenotype. The nicotine was administrated orally and dissolved in drinking water with 2% saccharin ad libitum [11,34,50]. Thus, two groups were created: prenatal nicotine + saccharin exposure (PNE) and prenatal saccharin exposure only (no−PNE).

### 5.3. Administration of MPH

A typical oral daily dose (0.5–2 mg/kg) of MPH in humans reaches a peak plasma concentration (2–15 ug/L) in 1–3 h, with a decay half-life of 1.5–2.5 h [51,52,53]. That dose exerts beneficial effects for 3–5 h, improving hyperactivity, inattention, and/or impulsivity symptoms. In mice, a 0.75 mg/kg oral dose produces within 15 min of administration, plasma levels of D-methylphenidate that are comparable to those seen in ADHD patients taking oral therapeutic doses of methylphenidate [54]. Therefore, behavioral tests were performed 75 min after MPH or vehicle administration. After this, some animals were sacrificed, and hippocampal slices were prepared for electrophysiological recordings (see below). Other animals were sacrificed 3 h after drug or vehicle administration to examine dendritic spines (see below).

Methylphenidate was administrated orally in a single dose of 1 mg/kg, following the protocol described by Zhang [55]. Briefly, the drug was delivered by using artificially flavored sweet jelly as a vehicle to avoid some additional stress factor in PNE mice. In the first step, the animals were trained to eat jelly. In the training step, an overnight starving period was applied, followed by the exposure to jelly, during a period of two days. At day 3, the drug was offered without previous starving.

### 5.4. Behavioral Tests

*Open Field Test.* The spontaneous motor activity of each experimental group was measured by open field test. For this purpose, the animals were placed in an open-field-recording device consisting of an acrylic box of 40 cm wide × 60 cm long × 40 cm high. Each animal was placed on the periphery of the box, and its trajectory was recorded for 10 min. The total distance traveled was recorded on video and measured by using the behavioral tracking software ANY-maze (Stoelting Company; IL, USA).

*Y-Maze Spontaneous Alternation Test.* The Y-maze spontaneous alternation paradigm is based on the natural tendency of rodents to explore a novel environment [56]. The arrangement consists of three opaque arms (each arm was 35 cm long × 6 cm wide × 10 cm high) that radiate from the center in a Y-shape. The behavioral test was initiated by placing the mouse in the center of the Y, which allows free access to the 3 arms for a period of 8 min, and recording was performed with an overhead video camera. An arm entry was counted when the four paws of the mouse entered the arm; the number of entries in each arm and the sequence of entries to the arms were monitored. A “Spontaneous Alternation” is defined as a set of choices of consecutive arms without a repeated entry. The spontaneous alternation score was calculated by using the following formula: number of alternations ÷ (number of total entries − 2) × 100. The behavioral data recorded were analyzed by using the behavioral tracking software ANY-maze (Stoelting Co., Wood Dale, IL, USA).

*Object-Based Attention (OBA) Test.* The test was performed by following the protocol of Alkam [57]. Briefly, the device consists of a rectangular box with two chambers: one for exploration and the other for testing (30 cm length × 30 cm width × 22 cm high, both). The experiments were divided into three phases: habituation, acquisition, and retention phases. In the habituation phase, the animals were individually subjected to a single session of habituation for 10 min, with exposure to both chambers, without objects. In the acquisition phase, the animals were exposed in a single session for 5 min to five objects (A, B, C, D, and E; made of the same material but with different forms) in the exploration chamber. In the retention phase, one object (A, for example) from the exploration chamber was transferred to the testing chamber, together with a novel object (F for example), and immediately after, the animal was exposed to the two objects (A and F) during 3 min in the testing chamber. All sessions were recorded, and the exploring time for each object was measured by using the ANY-maze software (Stoelting Co., Wood Dale, IL, USA). The recognition index for the retention phase was calculated as (TF × 100) / (TA + TF), where TA and TF are the exploring times for object A and for object F, respectively.

### 5.5. Hippocampal Slices

C57BL/6 mice were sacrificed by decapitation under halothane anesthesia. The brain was removed quickly and transferred into ice-cold solution containing (in mM) 125 NaCl, 4 KCl, 10 glucose, 1.25 NaH_2_PO_4_, 25 NaHCO_3_, 0.5 CaCl_2_, and 2.5 MgCl_2_ (pH 7.4) and equilibrated with a 5% CO_2_–95% O_2_ mixture. Hippocampi were cut in transversal slices of 300 μm with a vibratome (Leica, Nussloch, Germany). The slices were transferred to a storage chamber kept at room temperature in artificial cerebrospinal fluid (ACSF) containing (in mM) 124 NaCl, 2.5 KCl, 1.2 NaH_2_PO_4_, 24 NaHCO_3_, 5 HEPES, 12.5 glucose, 2 CaCl_2_, and 2 MgSO_4_ (pH 7.4, in 95% O_2_/5% CO_2_), and then they were kept for at least 1 h before the recordings. In the recording chamber, hippocampal slices were perfused with ACSF at a rate of 1 mL/min at RT.

### 5.6. Electrophysiology

Extracellular recordings in hippocampal slice were performed as described in Rozas et al. [19]. Field excitatory postsynaptic potentials (fEPSPs) were evoked by applying electrical stimulation delivered by an A360 stimulus isolator (WPInc, Sarasota, FL, USA), using bipolar concentric electrodes (200 μm diameter; FHC Inc., Bowdoinham, ME, USA) on Schaeffer collateral-commissural fibers and recorded with glass microelectrodes (1–2 MΩ) filled with ACSF from the stratum radiatum of the hippocampal CA1 region. Test pulses (0.2 ms) were applied every 15 s, and thecurrent was adjusted to evoke 50% of the maximal response. After recording a stable baseline for at least 20 min, LTP was induced by a theta burst stimulation (TBS, 5 trains of 10 bursts at 5 Hz each; 1 burst = 4 pulses at 100 Hz). In all experiments, the fEPSP recordings were maintained for 60 min after initiating TBS. The synaptic responses were quantified as the initial slope of fEPSPs and plotted as a percentage of change, referring to the initial slope measured during the baseline recording before TBS. To analyze presynaptic component of synaptic responses, a paired-pulse facilitation protocol was applied as follows: two pulses of 0.2 ms applied every 15 s, with an interstimulus interval of 50 ms applied before and after the TBS protocol. The results were presented as the ratio between the initial slopes of fEPSP evoked by the second stimulus and the first stimulus. This measure reflects the calcium-dependent quantal release of neurotransmitter from presynaptic components [58]. Excitatory Postsynaptic Currents (EPSCs) were recorded by using patch-clamp whole-cell recordings. CA1 neurons were visually identified with an infrared differential interference contrast microscope (Zeiss, Oberkochem, Germany). Patch pipettes (3–5 MΩ) were filled with internal solution containing (in mM) 130 Cs-gluconate, 2 ATP-Mg, 0.5 Na-GTP, 5 EGTA, 10 HEPES, and 1 QX-314, pH 7.4 (CsOH, 275–285 mOsm). Only cells with membrane potentials more negative than −65 mV, access resistance < 20 MΩ (8–18 MΩ, compensated at 80%), and input resistance > 100 MΩ (130–410 MΩ) were accepted for recordings. Bathing solution was ACSF supplemented with 10 μM picrotoxin in order to block GABAA-dependent currents. The AMPAR- and NMDAR-mediated EPSCs were recorded at holding potentials of −65 and +40 mV, respectively. NMDAR EPSC amplitude was measured 50 ms after the peak of AMPAR currents. In order to isolate the AMPAR EPSC, slices were perfused with ACSF containing 100 μM DL-2-amine-5-phosphopentanoic acid (DL-AP5). Rectification index of AMPAR EPSC was calculated as the ratio between the peak currents recorded at +40 mV and those recorded at −65 mV.

### 5.7. Western Blot

CA1 areas of hippocampal slices used in electrophysiological studies were dissected and homogenized in 200 µL of RIPA buffer containing 150 mM NaCl, 50 mM Tris-HCl, 5 mM EDTA, 5 mM EGTA, 50 mM NaF, 2.5 mM NaPP_i_, 1 mM Na_3_VO4, 1% NP-40, and 1% Na-deoxycholate, pH 7.4, supplemented with protease inhibitors (Halt™ Protease Inhibitor Cocktail, Thermo Scientific, Rockford, IL, USA). The homogenates were centrifuged at 12,000 rpm for 7 min at 4 °C, and the supernatants were collected and stored at −20 °C. Protein concentrations were determined by using the microBCA™ protein assay kit (Thermo scientific, Rockford, IL, USA). Then 50 μg of protein extracts was fractioned by PAGE in 12% polyacrylamide gels and transferred to nitrocellulose membranes for Western blot analysis. Primary antibodies were purchased from Millipore (Temecula, CA, USA) and used in the following dilutions: rabbit anti-GluA1 (1:500), rabbit anti-phospho-GluA1(Ser845) (1:1000), andrabbit anti-phospho-GluA1(Ser831) (1:1000). After incubation with HRP-conjugated secondary antibodies (1:10,000; Thermo Scientific, Rockford, IL, USA), reactive proteins were visualized by using chemiluminescent substrates (Thermo scientific, Rockford, IL, USA). GluA1 phosphorylation at Ser845 and Ser831 residues were quantified by using Image J software and normalizing the band intensity to the total GluA1-associated bands.

### 5.8. Crosslinking Assay

The crosslinking assay was performed as described in Rozas et al. [19]. Briefly, CA1 regions (taken from slices used in the electrophysiological experiments) were incubated with BS^3^ for 30 min at 4 °C. The crosslinked and non-crosslinked paired samples were prepared, and 40 μg of protein was charged and run in SDS–PAGE, using 4–15% gradient gels (BioRad, Hercules, CA, USA). The quantification of the intensity of bands and analyses were performed by using ImageJ software. The fraction of surface AMPA receptors in BS3-treated samples was estimated by densitometric measurement of the high-molecular-weight band associated with crosslinked receptors. The intracellular pool of receptors was estimated by measuring the density of bands associated to the monomeric non-crosslinked GluA1 subunit present in the same treated samples. The total amount of GluA1-containing AMPA receptors was estimated by densitometric measurement of the single band associated to monomeric GluA1 subunit present in the non-crosslinked paired samples for each experimental condition.

### 5.9. Golgi Staining and Dendritic Spines Analysis

Hippocampus slices used in electrophysiological recordings were collected, impregnated for 3 days, and then mounted and stained by using a Golgi stain kit (Bioenno Tech LLC, Santa Ana, CA, USA). The stained hippocampal neurons were examined by using confocal microscopy through Z-stacks of Golgi-stained neurons (up to 80 microns total on *z*-axis and an optical section; thickness = 0.5 µm), and photographs were taken at 63× magnification on a Zeiss AxioImager. On average, 12 Z-stacks were taken from each mouse. For density measurements, a minimum of 10 microns in dendrite length was taken into account, with dendritic spines visually distinct from one another having clearly defined spine heads. To estimate spine types, the geometry of the different spine shapes was examined to classify them with an unbiased method. In brief, measurements of the head width and neck length and the length–width ratio (LWR) were performed to determine spine types according to the following criteria: filopodia (length > 2 µm), long/thin (length > 1 µm), thin (LWR > 1 µm), stubby (LWR ≤ 1 µm), and mushroom (width > 0.6 µm) [59]. Analysis of density and morphology of dendritic spines was performed by using the Reconstruct free software (https://synapseweb.clm.utexas.edu/, accessed 10 December 2021).

### 5.10. Statistical Analysis

Statistical analysis was carried out by using Prism 6 software (GraphPad Software, SanDiego, CA, USA). Electrophysiological data are presented as mean ± SEM and are normalized relative to the baseline (average slope of fEPSPs measured before the TBS protocol). The (n,n) values displayed in the figures represent the number of animals and slices recorded, respectively. LTP was measured during the final 20 min of the recording and presented as the averaged percentage of baseline. For multiple comparisons, significance was determined by one or two-way ANOVA with Tukey’s post hoc test, where * *p* < 0.05, ** *p* < 0.01, *** *p* < 0.001, and **** *p* < 0.0001. For all statistical tests, normal distribution and variance of individual groups were considered similar.

## Data Availability

The data that support the findings of this study are available from the corresponding author upon reasonable request.
